# Supplementary material for: Fanconi Anaemia-Like Mph1 Helicase Backs up Rad54 and Rad5 to Circumvent Replication Stress-Driven Chromosome Bridges
Source: Genes (Basel). 2018 Nov 17;9(11):558. doi: 10.3390/genes9110558 (PMC6266064; doi:10.3390/genes9110558)
Supplement: Supplementary file 1 [file genes-09-00558-s001.pdf]

Fanconi anaemia-like Mph1 helicase backs up Rad54 and Rad5 to circumvent replication stress-driven chromosome bridges.

Jonay García-Luis[1,†] and Félix Machín[1,2*]

[1] *Unidad de Investigación, Hospital Universitario Nuestra Señora de Candelaria, Santa Cruz de Tenerife, Spain.*

[2] *Instituto de Tecnologías Biomédicas. Universidad de La Laguna, Santa Cruz de Tenerife, Spain.*

[†] *Present address: Cell Cycle Group, MRC Clinical Sciences Centre, Imperial College London, UK.*

[*] Correspondence should be addressed to Félix Machín, Ph. D.

Unidad de Investigación, Hospital Universitario Nuestra Señora de Candelaria, Carretera del Rosario, 145, 38010, Santa Cruz de Tenerife, Spain.

email: fmachin@funcanis.es

Supplementary Materials:

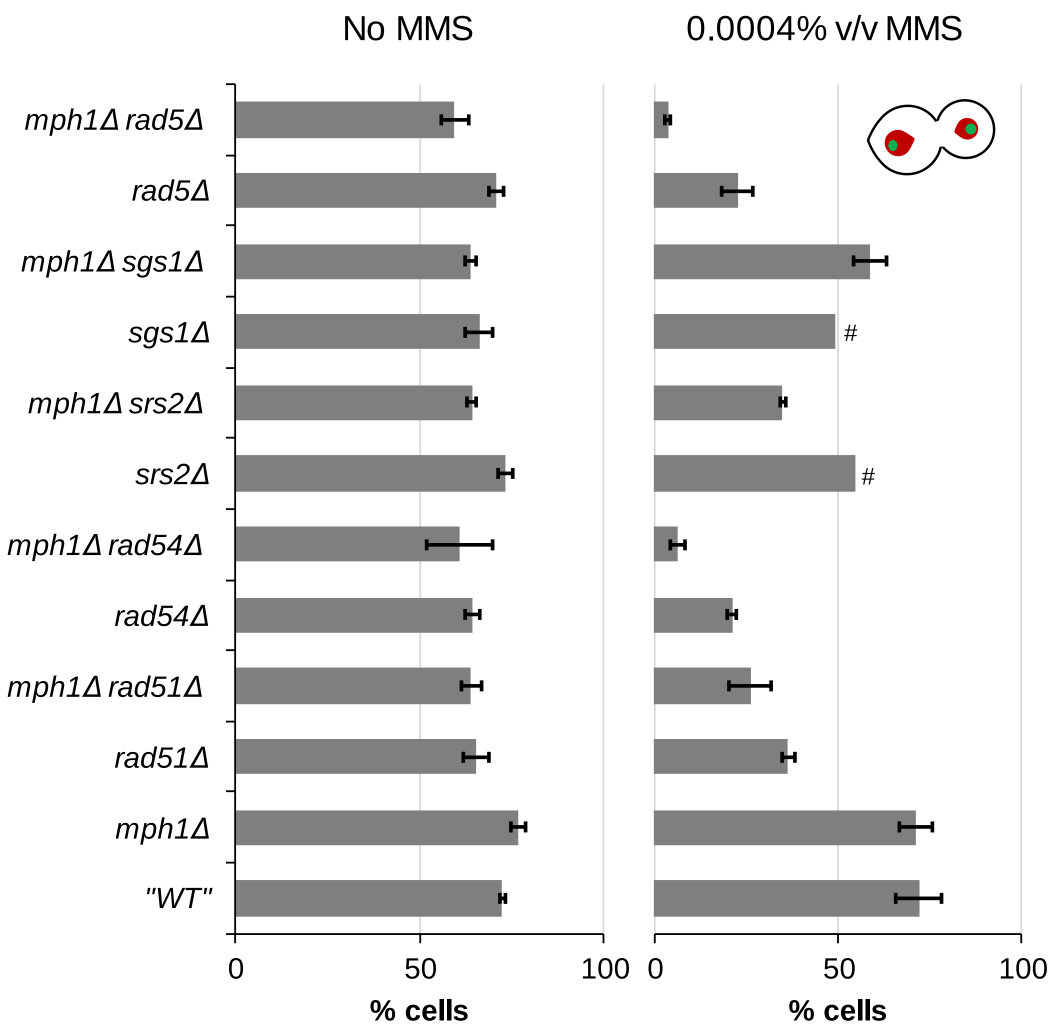

Figure S1. Completion of chromosome segregation under low exogenous replication stress for the helicase mutants included in this study. Data come from the main figures. To assess completion of chromosome segregation, *cdc15*-blocked binucleated cells with segregated Tel-cXIIr sister loci and no DAPI bridges (like in the schematic on the upper right) were plotted against all counted cells. The chart on the left depicts the situation without exogenous replication stress (4h after the G1 release to 37 ºC). The chart on the right depicts the situation under 0.0004% v/v MMS (borderline concentration for *rad* mutants, *rad mph1*Δ combinations and *srs2*Δ *mph1*Δ). The cell proportions are mean ± SEM (n=3); except for #, which indicates data coming from a single experiment.
